# Climate change and mental health: direct, indirect, and intersectional effects

**DOI:** 10.1016/j.lanepe.2024.100969

**Published:** 2024-06-14

**Authors:** Andreas Heinz, Lasse Brandt

**Affiliations:** a Department of Psychiatry and Neurosciences, Charité – Universitätsmedizin Berlin, Charité Campus Mitte, Corporate Member of Freie Universität Berlin, Humboldt Universität zu Berlin, and Berlin Institute of Health, Germany; b German Center for Mental Health (DZPG), partner site Berlin-Potsdam, Germany; c Bernstein Center of Computational Neuroscience Berlin, Germany; d Berlin School of Mind and Brain, Germany

Climate change is progressing rapidly and poses a threat to human physical and mental health. The human contribution to rising temperatures is well established, and data on the direct, indirect, and intersectional effects of climate change on mental health are increasing.^1^, ^2^, ^3^ This commentary highlights key aspects of the direct, indirect, and intersectional impacts of climate change on mental health.

Climate change is causing an increase in natural disasters, which can have a direct negative impact on mental health (Fig. 1).^1^, ^2^, ^3^ Rising temperatures are associated with increased mortality, morbidity, hospital admissions, and mental health burden for disorders such as suicide, anxiety, affective, and addictive disorders.^1^, ^2^, ^3^ According to a recent meta-analysis, at high ambient temperatures a 1 °C increase in temperature is associated with a 0.9% increase in mental health-related morbidity based on data from Asia, Oceania, Europe, and North and South America.^1^

**Fig. 1:**
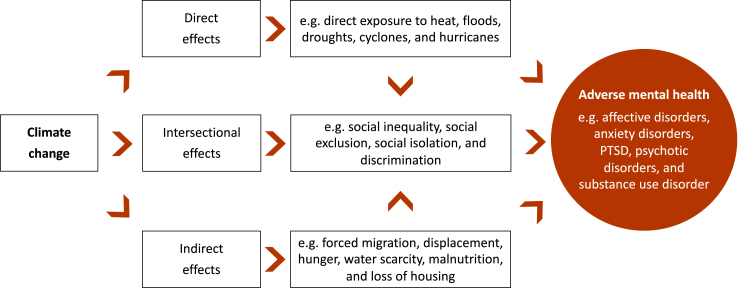
**Effects of climate change on mental health.** Climate change has direct, indirect, and intersectional effects on mental health. The direct, indirect, and intersectional effects of climate change are interconnected and can cause aggregate effects that are associated with increased mental health disorders. PTSD = Post-traumatic stress disorder.

Individuals exposed to natural disasters such as hurricanes, floods, and droughts are also at increased risk of mental disorders including post-traumatic stress disorder (PTSD), depressive disorders, and anxiety disorders.^1^, ^2^, ^3^ The severity and duration of mental disorders following natural disasters may be increased by psychosocial stressors such as personal and financial loss and forced migration, by vulnerabilities such as pre-existing mental disorders and low social support, and by insufficient mental health care.^2^ It is important to note that psychological and emotional responses to climate change may be appropriate considering the real threat of climate change, which should not be pathologised, but may also cause distress with anxiety and affective symptoms, currently discussed under terms such as “eco-anxiety” and “solastalgia”.^2^

Younger generations are affected by intergenerational inequality and carry a disproportionately higher burden of the effects of climate change compared with older generations.^2^^,^^4^ There is an urgent need to improve measures against climate change and support disproportionately affected groups including younger generations.^4^

Climate change may indirectly contribute to an increase in mental disorders through forced migration and displacement.^2^ For example, loss of living spaces due to inundation resulting from sea level rise and poverty are among climate-related drivers of forced migration and displacement.^2^ Forced migration is associated with a pronounced risk of mental disorders due to stressors before, during, and after migration.^5^ For example, the prevalence of substance use may be higher among individuals experiencing forced migration in camp settings, which are characterised by significant psychosocial challenges, compared to community settings.^6^ As another example, the stress of social isolation and discrimination may be increased for migrants in neighbourhoods with a lower proportion of individuals of similar origin, which is associated with an increased risk of psychosis.^7^

Importantly, climate change has intersectional effects on mental health, which can be defined as intersections between direct and indirect effects with an emphasis on social inequality and exclusion. Climate, health, and social inequalities intersect and vulnerable populations with lower socioeconomic resources, pre-existing mental disorders, and high exposure to climate related-environmental stressors may be at particular risk of experiencing increased health and social adversity.^8^^,^^9^ For example, individuals with lower socioeconomic status have a higher exposure to extreme heat and air pollution, are at increased risk of social isolation and discrimination, and have a higher risk of mental disorders, while individuals with higher socioeconomic status may be better protected against environmental stressors, experience less social adversity, and have lower barriers to access healthcare.^8^^,^^9^

Health, economic, social, regional, and intergenerational inequalities are anticipated to increase as a result of climate change.^2^^,^^8^ It is expected that costs, both in terms of health care costs and in terms of financial damage to economies, will increase. Mental health research requires increased funding to meet the direct, indirect, and intersectional challenges of climate change. According to advice from the European Scientific Advisory Board on Climate, countries should further develop robust climate change adaptation and mitigation policies, as well as disaster preparedness and response mechanisms.^10^ Disparities in access to resources and vulnerability between different socio-economic groups within European countries may exacerbate inequalities in mental health outcomes. European countries are part of a global community and should increase the collaboration on climate change mitigation and adaptation efforts through international agreements, research networks, funding programmes, and healthcare initiatives. This interconnectedness can facilitate knowledge sharing, resource mobilisation, and solidarity in addressing climate-related mental health challenges, both within Europe and in collaboration with other regions. For example, the European Psychiatric Association (EPA) has recently published a position paper on climate change and mental health and the newly established German Centre for Mental Health (DZPG) has initiated flagship research projects on important environmental topics such as urban mental health.^3^

## Contributors

AH and LB drafted the manuscript, critically revised the manuscript for important intellectual content, approved the final submitted version of the manuscript, and approved the decision to submit the manuscript.

## Declaration of interests

AH and LB are authors of the position papers of the European Psychiatric Association (EPA) and the German Association for Psychiatry, Psychotherapy and Psychosomatics (DGPPN) on climate change and mental health.
